# MEMS vibrational energy harvesters

**DOI:** 10.1080/14686996.2019.1569828

**Published:** 2019-02-18

**Authors:** Hiroshi Toshiyoshi, Suna Ju, Hiroaki Honma, Chang-Hyeon Ji, Hiroyuki Fujita

**Affiliations:** a Research Center for Advanced Science and Technology, The University of Tokyo, Tokyo, Japan; b Department of Electronic and Electrical Engineering, Ewha Womans University, Seoul, Korea; c Advanced Research Laboratories, Tokyo City University, Tokyo, Japan

**Keywords:** MEMS, microelectro-mechanical system, energy harvester, velocity-damped resonant generator, 60 New topics / Others, 201 Electronics / Semiconductor / TCOs, 206 Energy conversion / transport / storage / recovery, 208 Sensors and actuators, 400 Modeling / Simulations

## Abstract

In this paper, we look into the fundamental mechanism to retrieve the power from physical vibrations by using microelectromechanical systems (MEMS) energy harvesters. An analytical model is presented for the velocity-damped resonant generator (VDRG) that delivers electrical power through the power enhancement mechanism using the mechanical resonance of a suspended mass. Deliverable power is also analytically discussed with respect to the theoretical limit, and a view to understand the VDRG behaviors is presented in association with the impedance matching condition and the quality factors. Mechano-electric power conversions including electrostatic induction, electromagnetic induction, and piezoelectric effect are discussed to study the scaling effect. Recent examples of MEMS VDRGs are reviewed and evaluated in terms of the power density.

## GRAPHICAL ABSTRACT

**Figure:**
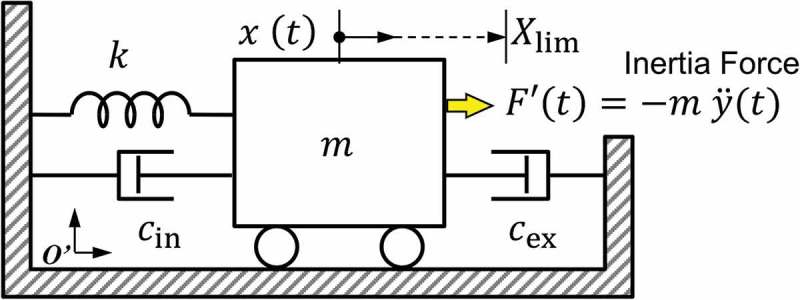

## 1. Introduction

Remarkable progress in digital information processing and communication has enabled the smart society where everything is connected in a network. The network society is defined as ‘a society in which a combination of social and media networks shapes its prime mode of organization and most important structures at all levels from individual to organizational and societal’ [1]. Nowadays, the level of connection has been further extended to environmental and machinery. What happens in the physical world is automatically reflected in the cyber world through the communication network or so-called the Internet of Things (IoT). In the IoT system, sensing nodes, like our sensory organs, play the interfacing role between the physical and cyber worlds. The sensing node has four basic functions, namely, sensing, signal/information processing, communication, and power supply.

Microelectromechanical systems (MEMS) is the most suitable technology to realize IoT-sensing nodes because it enables integrated fabrication of sensors/actuators, electronic circuits for information processing and radio frequency communication, antennas, and energy harvesters on a single chip or in a package [2]. Integrated sensors and low-power electronics are well developed among those functions. For example, Pruitt et al. fabricated multifunctional integrated sensors that combine temperature, humidity, pressure, air speed, chemical gas, magnetic, and acceleration sensing on a single 2-mm × 2-mm die [3]. Peak power consumption of IoT wireless sensors are tens of mW as shown in Table 1; however, average power consumption can be tuned to a 100 µW by reducing the duty ratio for wireless communication through the intermittent operation, thereby allowing energy harvesters as a power source for them.

**Table 1.** Typical power demand of recent microelectronics.

Device	Power	Current	Voltage	Note
ZigBee	60 mW peak	20 mA	3.0 V	IEEE 802.15.4, datasheet^a^
Apple Watch	52 mW avg	13.8 mA	3.76 V	Battery 250 mAh for 18 h by official benchmark protocol^b^
Bluetooth 4.0 (BLE)	45 mW peak	15 mA	2.0 ~ 3.6 V	Manufacturer datasheet^c^
GPS Tracker (BLE)	↑ (intermittent)	↑ (intermittent)	3.0 V	CR2016 (90 mAh) for 1 year^d^
Felica (readout)	~ 15 mW peak	~ 5 mA	3.0 V	Manufacturer datasheet^e^
Hearing Aid	~ 1 mW avg	0.67 mA	1.4 V	PR536 Battery 100 mAh for 150 h
Heart Pace Maker	33 µW avg	13 µA	2.5 V	Battery 1.15 Ah for 10 years^f^
Analog Clock LSI (Wrist Watch)	2.8 µW avg	1.0 µA	2.8 V	Manufacturer datasheet^g^
	0.39 µW avg	0.25 µA	1.55 V	Manufacturer datasheet^h^
Timer IC	88 nW avg	35 nA	2.5 V	Manufacturer datasheet^i^

^a^ZigBee (Zigbee Alliance, USA) Wireless Networking Overview (Rev. D), Texas Instruments Inc. (USA) [4]. ^b^Apple Watch Series 3 Battery Information, Apple Inc. (USA) [5]. ^c^Bluetooth Low Energy, Lapis Semiconductor Co. Ltd. (Japan) [6]. ^d^GPS Tracker XY4+, XY– The Findables Company (USA) [7]. ^e^Felica card reader, nocoly Inc. (Japan) [8]. ^f^Hearing aid, Advisa MRI (A3DR01), Medtronic PLC (USA) [9]. ^g^Clock LSI, SEIKO NPC Corp (Japan) [10]. ^h^Timer IC TPL5010/TPL5110, Texas Instruments Inc. (USA) [11].

However, there are some elements to be further improved. Among such missing elements, the energy source is the most crucial issue. One of the most terrified moments in our daily life is a situation with a low-power alarm in the middle of an urgent business talk on a cellular phone, as it depends solely on a capacity of the battery, and so do most current IoT nodes. Unlike commercial cellular phones that allow us to change the power packs at will, those IoT applications including logistics, environmental monitoring, industrial plant management, and infrastructure integrity monitoring would have uneconomical difficulties in regular exchanging the batteries. It is therefore desirable to use the energy harvested from the environment for such IoT nodes, like small living creatures feeding themselves all the time.

After many years of development of energy harvesters, more than a few mW of power can be gained for a practical use of electronics by using photovoltaic solar cells in open fields and thermoelectric generators attached to high-temperature structures and by proximity wireless power transmission for radio frequency identification. Nonetheless, their usage is still limited to a fraction of potential applications because they require specific place and/or time to work efficiently. Therefore, alternative or supplemental energy source should be utilized in order to widen the applicable fields and situation of the IoT nodes. Mechanical vibration is an attractive candidate because it exists almost everywhere at any time. The conversion mechanisms from the motion of a mass to electrical power include electromagnetic [12], piezoelectric [13], triboelectric [14], and electrostatic [15,16]. All those devices can produce electrical power ranging from a few tens of µW to hundreds μW. However, there are still remaining issues including (1) further reduction of device dimensions, (2) matching with the environmental vibration frequencies which may vary on location and time, (3) device improvement toward larger power and higher energy conversion rate, (4) integration capability in terms of processing and materials, and (5) mass manufacturability.

Here, we will discuss the latest development of MEMS vibrational energy harvesters toward solving these issues. Examples include electrostatic devices, electromagnetic, and piezoelectric devices. Our major aim of the review is to provide the reader with the theoretical analysis of the fundamental behavior based on a simplified model and to provide with insights for optimization and comparison of the performance, such as deliverable power and effectiveness of energy harvesters.

## 2. Theory of vibrational energy harvester

### 2.1. Coupled resonator model

The target of this study is to integrate the mechanical and electrical analytical models for vibrational energy harvesters to comprehend the power conversion mechanism from an impedance-matching point of view of electrical circuit. We presume a resonant type harvester whose mechanical resonant frequency is tuned to the prominent frequency of the environmental vibrations. Such a mechanism is usually referred to as the velocity-damped resonance generator (VDRG) [17], as the velocity of a physically excited mass is mechano-electrically coupled to deliver electrical power to the external load.

**Figure 1.**
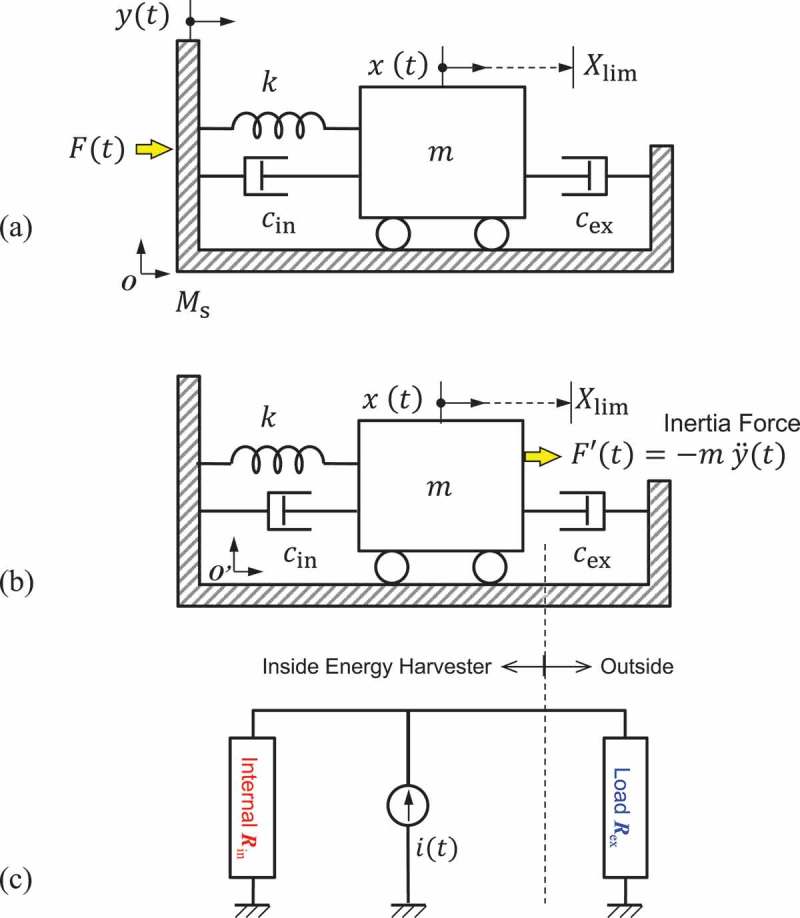
Analytical model for the velocity damped resonance generator (VDRG). (a) Initial model in an inertia system *O*, where the shell mass $M_{s}$ should be considered to indirectly excite the suspended mass m. (b) Simplified model in a non-inertia frame *O*ʹ, where the suspended mass is driven by the inertia force. (c) Equivalent circuit model with a current source.

The analytical model for the VDRG is represented by using a two-mass model illustrated in Figure 1(a). The internal mass m is suspended with a suspension of a spring constant k and with two parallel dashpots. The first dashpot on the left with a viscous damping coefficient $c_{in}$ represents the internal loss that consumes power within the energy harvester, while the second one on the right with another coefficient $c_{ex}$ represents the output power that can be taken out of the harvester to an external load. The internal mass is anchored to the outer shell of mass $M_{s}$, which is directly shaken by the external force $F\left(t\right)$ at an amplitude of $y\left(t\right)$ as a function of time t.

However, the two-mass model in the inertia system O in Figure 1(a) is complicated, as it urges us to use the two degrees of freedom $x\left(t\right)$ and $y\left(t\right)$ as coupled resonators. Also, due to the practical reason that the excitation force $F\left(t\right)$ is not usually explicitly measured on the harvester package, unless we deliberately insert a force sensor in between the excitation source and the harvester device without causing disturbance, the excitation amplitude $y\left(t\right)$ is the only known input to the system. In addition to this, the mass of the shell $M_{s}$ is not be uniquely defined by the mass of the harvester package alone, particularly when the harvester is fixed onto a printed circuit board (PCB), for instance. In such a case, the shell mass $M_{s}$ varies due to the masses of other components added onto the PCB that is mechanically shaken as one. Therefore, we do not use the shell mass $M_{s}$ in this analysis but we exclude it by converting the initial model in the inertia system O in Figure 1(a) into a simplified model in the non-inertial frame $O{\;}^{\prime }$ as illustrated in Figure 1(b).

In the non-inertial frame, the equation of motion of the suspended mass is simply written as

(1) $$m\;\ddot{x}\left(t\right)+c\;\dot{x}\left(t\right)+k\;x\left(t\right)=F{\;}^{\prime }\left(t\right),$$

where $F{\;}^{\prime }\left(t\right)=-m\;\ddot{y}\left(t\right)$ is the inertia force acting on the mass [18]. The total viscous damping coefficient c is the sum of the internal loss $c_{in}$ and the external loss $c_{ex}$ as

(2) $$\begin{array}{c} c=c_{in}+c_{ex}. \end{array}$$

These two components are understood by the Joule heat dissipation in the internal and external electrical resistances in the equivalent circuit model shown in Figure 1(c), where a current source is used as a power source. The power consumed in the external load resistance $R_{ex}$ corresponds to that dissipated in the external load $c_{ex}$, while the internal resistance $R_{in}$ is associated to the power consumption in the internal loss $c_{in}$. Note that the internal loss $c_{in}$ includes both the mechanical loss caused by the air viscosity and the electrical loss caused by the internal electrical resistance of the harvester; neither of them is retrieved to outside but they are consumed within the harvester device. Therefore, they should be handled separately from the deliverable power to the external load resistance $R_{ex}$. The behavior of generator can also be equivalently understood by using a voltage source with serially connected internal and external resistances. However, we use a current source model in this work because the resistances can be arranged in a parallel format on both sides of the power source as shown in Figure 1(c), which is visually more readable to correlate with the internal and external dashpots in Figure 1(b).

Assuming a harmonic analysis at the angular frequency ω, we write the excitation force as

(3) $$\begin{array}{c} F{\;}^{\prime }\left(t\right)=F_{0}^{{\;}^{\prime }}\exp\left(i\omega t\right), \end{array}$$

where $F_{0}^{{\;}^{\prime }}$ is the peak value of the force. The oscillation of the suspended mass will have a similar form of a sinusoidal response but with a phase delay ϕ as

(4) $$\begin{array}{c} x\left(t\right)=A\left(\omega \right)\exp\left\{i\left(\omega t-\phi \right)\right\}, \end{array}$$

where $A\left(\omega \right)$ is the amplitude of the oscillation. The first- and the second-order differentials of $x\left(t\right)$, are respectively, written as $\dot{x}\left(t\right)=i\omega x\left(t\right)$ and $x\left(t\right)=-\omega^{2}x\left(t\right)$. By substituting these into Equation (1), we obtain

(5) $$\begin{array}{rl} & \left(-m\;\omega^{2}+i\;c\;\omega +k\right)\;A\left(\omega \right)\\ & \;\exp\left\{i\left(\omega t-\phi \right)\right\}=F_{0}^{{\;}^{\prime }}\exp\left(i\omega t\right). \end{array}$$

Therefore, the phase component is written as

(6) $$\begin{array}{r} e^{-i\phi }=\frac{F_{0}^{{\;}^{\prime }}}{A}\cdot \frac{1}{-m\;\omega^{2}+i\;c\;\omega +k}. \end{array}$$

By taking the norm of this equation, we write

(7) $$\begin{array}{r} \left|e^{-i\phi }\right|=\frac{F_{0}^{{\;}^{\prime }}}{A}\cdot \frac{1}{\sqrt{{\left(k-m\;\omega^{2}\right)}^{2}+c^{2}\;\omega^{2}}}=1. \end{array}$$

Therefore, the amplitude of oscillation $A\left(\omega \right)$ is found as a function of the angular frequency as

(8) $$\begin{array}{r} A\left(\omega \right)=\frac{F_{0}^{{\;}^{\prime }}}{\sqrt{{\left(k-m\;\omega^{2}\right)}^{2}+c^{2}\;\omega^{2}}}. \end{array}$$

By comparing the real and the imaginary parts of Equation (6), the phase is also derived as

(9) $$\begin{array}{r} \phi \left(\omega \right)=tan^{-1}\frac{c\;\omega }{k-m\;\omega^{2}}. \end{array}$$

From Equation (8), one would see that the amplitude $A\left(\omega \right)$ takes its maximum when the denominator becomes minimum. By differentiating $B={\left(k-m\;\omega^{2}\right)}^{2}$ $\;+c^{2}\;\omega^{2}$ with respect to $\omega^{2}$, we obtain

(10) $$\begin{array}{r} \frac{dB}{d\left(\omega^{2}\right)}=-2\;m\;\left(k-m\;\omega^{2}\right)+c^{2}. \end{array}$$

The stationary condition for Equation (10), i.e.,

(11) $$\begin{array}{r} \omega_{0}=\sqrt{\frac{k}{m}-\frac{c^{2}}{2m^{2}}} \end{array}$$

is usually referred to as the resonant (angular) frequency, which shifts slightly toward the lower frequency as the viscous damping coefficient c increases. When the viscous damping is virtually ignored, the oscillation system resonates at the eigen frequency (or natural frequency)

(12) $$\begin{array}{r} \omega_{n}=\sqrt{\frac{k}{m}}. \end{array}$$

For simplicity, we hereafter use the normalized angular frequency $\omega_{c}$ with respect to the eigen frequency as

(13) $$\begin{array}{r} \omega_{c}=\frac{\omega }{\omega_{n}}. \end{array}$$

**Figure 2.**
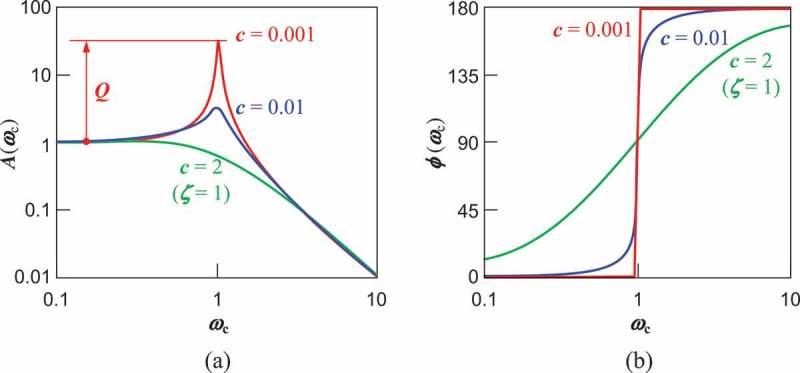
Frequency responses of the oscillator angle and phase calculated at different values of damping. Frequency is normalized to the eigen frequency.

Figure 2(a,b), respectively, shows the oscillation amplitude $A\left(\omega_{c}\right)$ and the phase $\phi \left(\omega_{c}\right)$ as a function of the normalized angular frequency. A VDRG is usually designed to have a small mechanical loss to extract a large output power from a resonating mass whose amplitude is *Q*-fold enhanced from the static displacement. In the resonance condition, the oscillation $x\left(t\right)$ is 90° behind the excitation force $F^{\prime }\left(t\right)$. The velocity $\dot{x}\left(t\right)$, which is 90° ahead of $x\left(t\right)$, is thus in phase with the force. The mechanical work performed by the force, $\int F{\;}^{\prime }\left(t\right)\;\dot{x}\left(t\right)\;dt$, is a non-zero value, and therefore the oscillating body gains energy from the excitation force.

From Equation (8), the amplitude can be rewritten by using the normalized frequency $\omega_{c}$ as

(14) $$\begin{array}{r} A\left(\omega_{c}\right)=\frac{F_{0}^{{\;}^{\prime }}}{k}\cdot \frac{1}{\sqrt{{\left(1-{\omega_{c}}^{2}\right)}^{2}+4\;\zeta^{2}{\omega_{c}}^{2}}}, \end{array}$$

where $\zeta =c/\left(2m\omega_{n}\right)=c/\left(2\sqrt{m\;k}\right)$ is the viscous damping ratio, which is an alternative expression of the viscous damping coefficient in the normalized equation of motion $\ddot{x}+2\zeta \omega_{n}\dot{x}+\omega_{n}^{2}x=F{\;}^{\prime }\left(t\right)/m$. In Equation (14), the first factor $F_{0}^{{\;}^{\prime }}/k$ represents a virtual static extension of the spring k when a static force of $F_{0}^{{\;}^{\prime }}$ is applied, and hence the rest of the equation is thought to be an amplitude magnification factor M with respect to the static displacement $F_{0}^{{\;}^{\prime }}/k$ as

(15) $$\begin{array}{r} M\left(\omega_{c}\right)=\frac{1}{\sqrt{{\left(1-{\omega_{c}}^{2}\right)}^{2}+4\;\zeta^{2}{\omega_{c}}^{2}}}. \end{array}$$

At the resonance ($\omega_{c}=1$), the amplitude magnification factor is referred to as the quality factor or Q as

(16) $$\begin{array}{r} Q=\frac{1}{2\zeta }=\frac{\sqrt{m\;k}}{c}. \end{array}$$

As the total viscous damping coefficient c includes the contribution from the internal dashpot as well as the external one, the total quality factor is also rewritten by using these elemental losses as

(17) $$\begin{array}{r} Q^{-1}=Q_{in}^{-1}+Q_{ex}^{-1}, \end{array}$$

where $Q_{in}=\sqrt{m\;k}/c_{in}$ and $Q_{ex}=\sqrt{m\;k}/c_{ex}$. These two terms are frequently used to discuss the power leverage factor and the impedance matching ratio of the energy harvester in the subsequent sections.

### 2.2. Mechano-electric power conversion

The output power of the VDRG [17] is modelled by the power dissipation of the external dashpot $c_{ex}$ in Figure 1(b). The viscous resistance acting on the dashpot is written as $c_{ex}\cdot \dot{x}\left(t\right)$, where $\dot{x}\left(t\right)$ is the velocity of the suspended mass. The power (or the mechanical work performed per second) is the product of the viscous resistance and the velocity,

(18) $$P\left(t\right)=c_{ex}\cdot \dot{x}\left(t\right)\cdot \dot{x}\left(t\right)=c_{ex}\cdot \dot{x}{\;}^{2}\left(t\right).$$

By differentiating Equation (4) with respect to time, the velocity is written as

(19) $$\dot{x}\left(t\right)=i\;\omega \;A\left(\omega_{c}\right)\exp\left\{i\left(\omega t-\phi \left(\omega \right)\right)\right\},$$

therefore

(20) $${\dot{x}}^{2}\left(t\right)=-\omega^{2}A{\left(\omega_{c}\right)}^{2}\;\exp\left\{2i\left(\omega t-\phi \left(\omega \right)\right)\right\}.$$

The peak value of ${\dot{x}}^{2}\left(t\right)$ is $\omega^{2}A^{2}$, and therefore its effective value is $\omega^{2}A^{2}/2$. Replacing ${\dot{x}}^{2}\left(t\right)$ in Equation (18) with $\omega^{2}A^{2}/2$, we obtain the expression of the effective output power $P_{rms}$ as

(21) $$\begin{array}{rl} & P_{rms}=\frac{1}{2}c_{ex}\;\omega^{2}A^{2}\left(\omega_{c}\right)\\ & \;\;=\frac{1}{2}c_{ex}\;\omega^{2}{\left(\frac{F_{0}^{{\;}^{\prime }}}{k}\right)}^{2}\frac{1}{{\left(1-{\omega_{c}}^{2}\right)}^{2}+4\zeta^{2}{\omega_{c}}^{2}}, \end{array}$$

where the amplitude $A\left(\omega_{c}\right)$ is replaced by Equation (14). Here, we remember that the inertia force in Equation (3) is also written as

(22) $$F{\;}^{\prime }\left(t\right)=F_{0}^{{\;}^{\prime }}\exp\left(i\omega t\right)=-m\;\ddot{y}\left(t\right),$$

and then we rewrite the excitation acceleration, velocity, and displacement respectively as

(23a) $$\ddot{y}\left(t\right)=-\frac{F_{0}^{{\;}^{\prime }}}{m}\exp\left(i\omega t\right),$$

(23b) $$\dot{y}\left(t\right)=-\frac{F_{0}^{{\;}^{\prime }}}{i\omega m}\exp\left(i\omega t\right),$$

(23c) $$y\left(t\right)=\frac{F_{0}^{{\;}^{\prime }}}{\omega^{2}m}\exp\left(i\omega t\right)=y_{0}\exp\left(i\omega t\right),$$

where $F_{0}^{{\;}^{\prime }}/\left(\omega^{2}m\right)$ is now defined as $y_{0}.$ We replace the force $F_{0}^{{\;}^{\prime }}$ with $\omega^{2}m\;y_{0}$ in Equation (21), and the effective power is further rewritten as

(24) $$\begin{array}{rl} & P_{rms}=\frac{1}{2}c_{ex}\;\omega^{2}{\left(\frac{1}{k}\right)}^{2}\frac{\omega^{4}m^{2}\;y_{0}^{2}}{{\left(1-{\omega_{c}}^{2}\right)}^{2}+4\zeta^{2}{\omega_{c}}^{2}}\\ & \;\;\;\;\;=\frac{1}{2}c_{ex}\;\frac{\omega^{6}}{\omega_{n}^{4}}\cdot \frac{y_{0}^{2}}{{\left(1-{\omega_{c}}^{2}\right)}^{2}+4\zeta^{2}{\omega_{c}}^{2}}. \end{array}$$

The external loss $c_{ex}$ is also expressed by using a damping ratio $\zeta_{ex}$ as

(25) $$c_{ex}=2\;m\;\zeta_{ex}\;\omega_{n},$$

therefore, the effective power at the angular frequency ω is finally expressed as

(26) $$P_{rms}=\frac{1}{2}\cdot \frac{2\;m\;\zeta_{ex}\;\omega_{c}^{3}\;\omega^{3}\;y_{0}^{2}}{{\left(1-{\omega_{c}}^{2}\right)}^{2}+4\zeta^{2}{\omega_{c}}^{2}},$$

where ζ is the overall damping ratio of the system that includes both the internal and external losses as

(27) $$\zeta =\zeta_{in}+\zeta_{ex}.$$

The individual damping ratios are also associated to the quality factors by $\zeta_{in}=1/\left(2\;Q_{in}\right)$ and $\zeta_{ex}=1/\left(2\;Q_{ex}\right)$. Equation (26) still includes the angular frequency ω as a hidden variable in $\omega_{c}$, but we leave it as is for the convenience in the following discussion.

### 2.3. Output power at resonance

As derived by Mitcheson et al. [17], the output effective power in Equation (26) is simplified to

(28) $$P_{rms}=\frac{m\;\zeta_{ex}\;\omega_{n}^{3}\;y_{0}^{2}}{4\zeta^{2}},$$

when the VDRG is operated at its resonant frequency $\omega_{c}=1$, hence $\omega =\omega_{n}.$

By using the components of energy loss (Equations (17) and (27)), the effective power is further modified to

(29) $$\begin{array}{rl} & P_{rms}=\frac{m\;\zeta_{ex}\;\omega_{n}^{3}\;y_{0}^{2}}{4\;{\left(\zeta_{in}+\zeta_{ex}\right)}^{2}}\\ & \;\;=\frac{1}{2}m\;\omega_{n}^{3}\;y_{0}^{2}\cdot Q\cdot \frac{1}{1+\frac{Q_{ex}}{Q_{in}}}\\ & \;\;=\frac{1}{2}m\;\omega_{n}^{2}\;y_{0}\cdot \omega_{n}\;y_{0}\;Q\cdot \frac{1}{1+\frac{Q_{ex}}{Q_{in}}}. \end{array}$$

Remembering that ${\omega_{n}}^{2}\;y_{0}$ is the peak excitation acceleration applied to the harvester at resonance, the term $m\;{\omega_{n}}^{2}\;y_{0}$ is the peak value of the inertia force acting on the suspended mass. In addition to this, $\omega_{n}\;y_{0}\;Q$ is the peak velocity of the suspended mass that has been *Q*-fold amplified from the excitation peak velocity $\omega_{n}\;y_{0}$. The force and the velocity are in phase at the resonance, and their product represents the finite effective mechanical power incoming to the harvester as

(30) $$P_{in}=\frac{1}{2}m\;\omega_{n}^{2}\;y_{0}\cdot \omega_{n}\;y_{0}\;Q.$$

In other words, the oscillating mass would have received small power of $m\;\omega_{n}^{3}\;{y_{0}}^{2}/2$ if it were directly excited by the external force that gives an amplitude of $y_{0}$. By using the resonance mechanism, on the other hand, the oscillating amplitude and hence the velocity of the mass is *Q*-times enhanced, and so is the output power. The rest of Equation (29) $1/\left(1+Q_{ex}/Q_{in}\right)$ is the impedance matching ratio, which is discussed in Section 2.4.

### 2.4. Q-factor dependence

**Figure 3.**
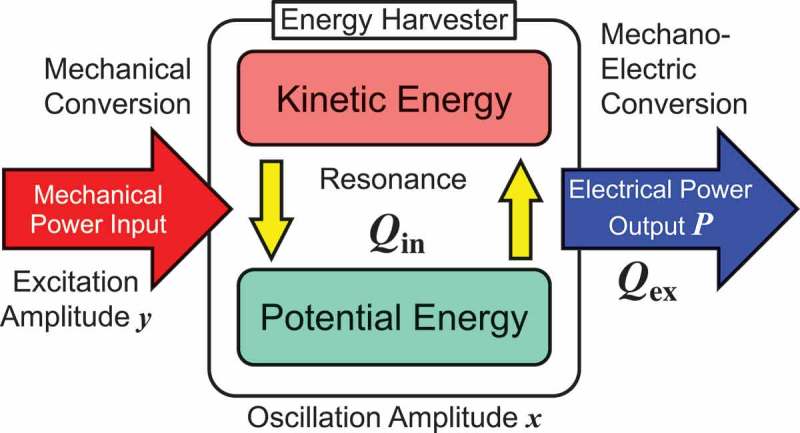
Schematic diagram of the power flow through a VDRG.

Power conversion mechanism is understood by using the schematic diagram shown in Figure 3. An oscillating body at resonance is an energy transduction system that reciprocally exchanges energy between the kinetic energy of the moving mass and the potential energy of the spring at an efficient rate determined by the quality factor $Q_{in}$. Due to the negligibly small power loss in this stage, the overall power conversion efficiency is governed by the effectiveness of the mechano-electric conversion at the output stage, which is described by the term ${\left(1+Q_{ex}/Q_{in}\right)}^{-1}$ that could be found in Equation (29).

To further study the power conversion efficiency and the effectiveness by using Equation (29), we extract the following two factors as

(31) $$\chi =\frac{1}{\frac{1}{Q_{in}}+\frac{1}{Q_{ex}}}\;\frac{1}{1+\frac{Q_{ex}}{Q_{in}}},$$

(32) $$\eta =\frac{1}{1+\frac{Q_{ex}}{Q_{in}}}.$$

In this work, we call χ as the power leverage factor at resonance, as it determines the magnifying factor for the output power with respect to the deliverable power when no resonance is presumed. The latter factor η is called the impedance matching ratio that determines the ratio of the output power to the total power. In addition to this, we define the power recovery effectiveness that determines the ratio of the output power with respect to the theoretical maximal value as

(33) $$E_{H}=\frac{P_{exp}}{P_{rms}},$$

where $P_{exp}$ is the experimentally obtained output power and $P_{rms}$ is the theoretically expected output defined by Equation (29).

**Figure 4.**
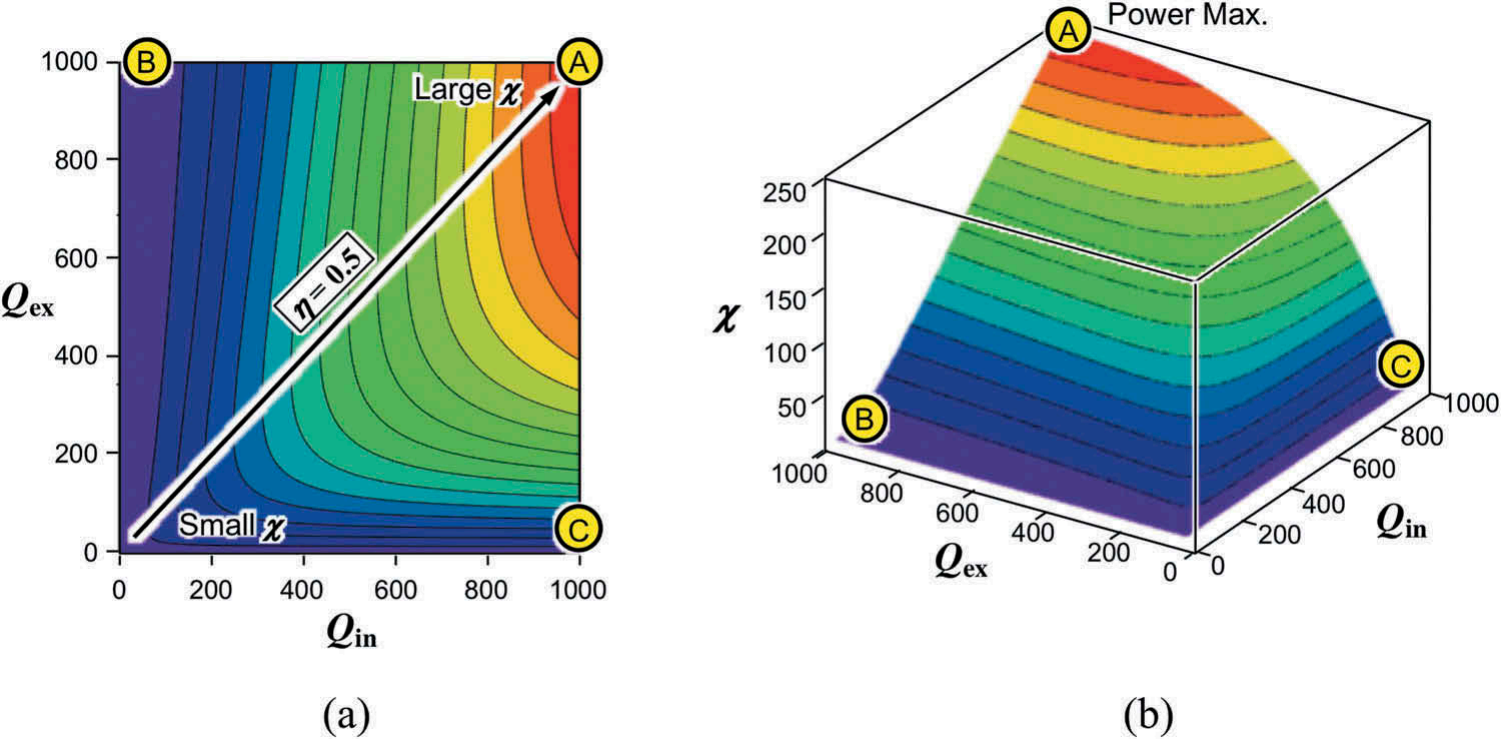
Power leverage factor χ calculated as a function of the internal and external quality factors when the oscillation is not limited. (a) Contour plot and (b) 3D plot. Output power is enhanced when $Q_{in}$ and $Q_{ex}$ are equally maximized.

Figure 4(a) plots the contour map of the power leverage factor χ as a function of the internal quality factor $Q_{in}$ and the external quality factor $Q_{ex}$. The same plot is extruded into the three-dimensional view as shown in Figure 4(b). As we call χ the power leverage factor, it naturally takes values more than unity. Given the mass m, the resonant frequency $\omega_{n}^{3}$, and the excitation amplitude $y_{0}$, one would pursue the maximum power output at point A, where the internal and the external quality factors are equally enlarged to their limit, $Q_{in}=Q_{ex}$. When $Q_{in}\ll Q_{ex}$ as seen at point B, the mechanical resonance is not efficiently excited due to the large internal loss (or small $Q_{in}$). At the same time, the oscillation cannot be converted into the electrical output due to the small mechano-electric coupling (or high $Q_{ex}$). When $Q_{in}\gg Q_{ex}$ at point C, on the other hand, the amplitude of the mass can be potentially excited to a large value due to the small internal loss (or high $Q_{in}$) if no electrical output is connected. However, due to the presence of relatively large external loss (or low $Q_{ex}$), the amplitude remains small, thereby delivering small output power.

**Figure 5.**
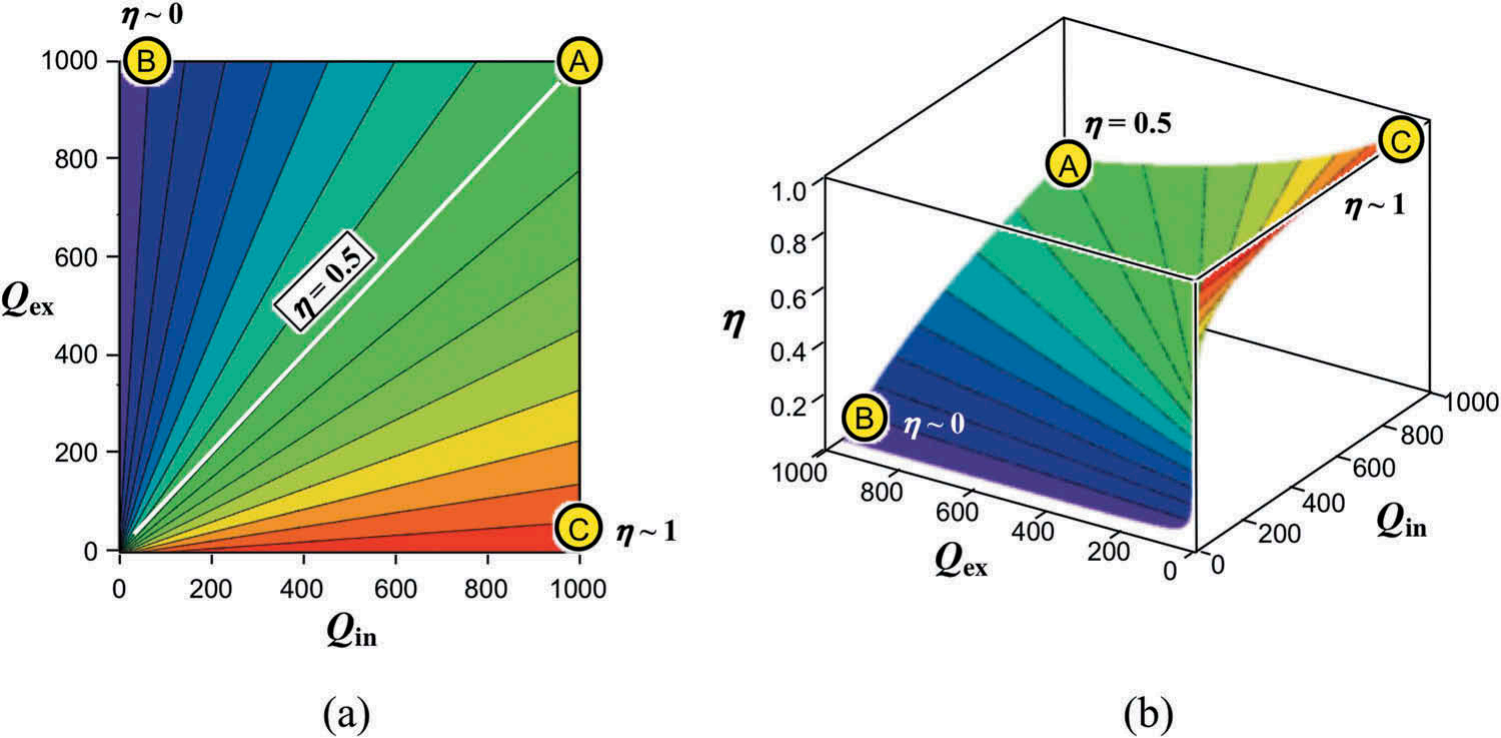
Impedance matching ratio η calculated as a function of the internal and external quality factors. (a) Contour plot and (b) 3D plot. Output power becomes 50% of the total source power when $Q_{in}=Q_{ex}$. Note that a high η does not always yield large output power when cross-referenced with χ in Figure 4.

The impedance matching ratio η is calculated and presented in a similar display format in Figure 5(a,b). The profile looks like a spiral staircase, and it yields small values whenever the internal quality factor $Q_{in}$ is low, while it reaches almost the theoretical limit of η=1 when the external quality factor $Q_{in}$ becomes low, implying that all the mechanical incoming power gained by the resonance is effectively converted into the electrical output power; nonetheless, the absolute output power might stay low as seen in Figure 4(b). From a deliverable power point of view, the condition at point A should be the best solution as it gives the theoretical maximum power at an effectiveness of 50%.

### 2.5. Analogy to equivalent electrical circuit model

The physical implications of χ and η can be intuitively understood by using the analogy to describe the equivalent electrical circuit shown in Figure 1(c). The current source $i\left(t\right)$ delivers electrical power to the internal resistance $R_{in}$ and the external one $R_{ex}$. The total parallel resistance seen from the current source is written as $R=R_{in}R_{ex}/\left(R_{in}+R_{ex}\right)$, and the voltage commonly applied to the resistances is accordingly calculated to be R⋅i. The electrical powers dissipated in the external resistance is therefore

(34) $$\begin{array}{rl} & P_{ex}=\frac{1}{R_{ex}}\;{\left(\frac{R_{in}\;R_{ex}\;i}{R_{in}+R_{ex}}\right)}^{2}\\ & \;\;\;=\frac{i^{2}}{\frac{1}{R_{in}}+\frac{1}{R_{ex}}}\;\frac{1}{1+\frac{R_{ex}}{R_{in}}}. \end{array}$$

**Figure 6.**
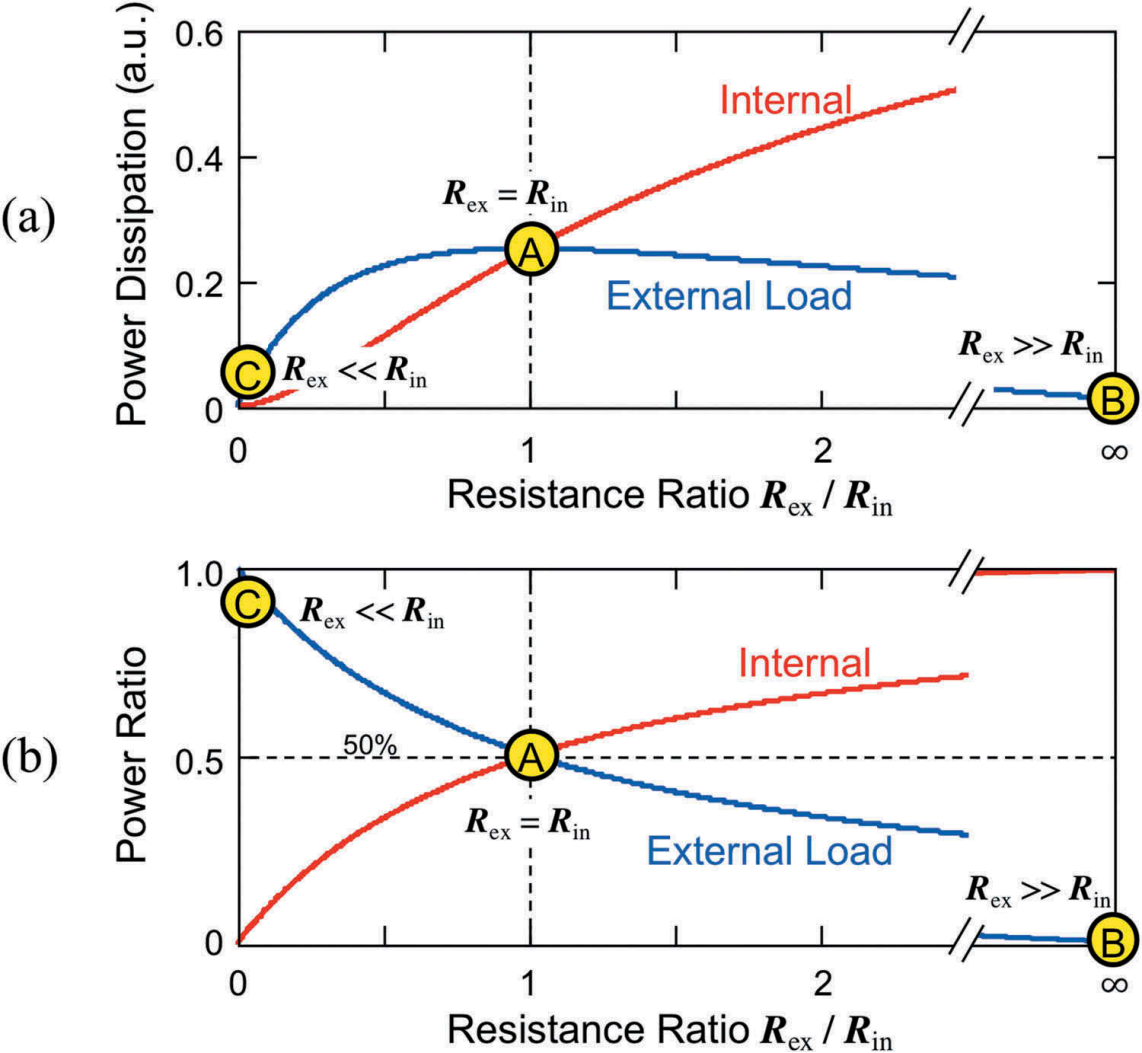
Deliverable power from an equivalent current source (shown in Figure 1(c)). (a) Power dissipation in the internal and external resistances. (b) Power allocation ratio. Maximum power is delivered when $R_{ex}=R_{in}$ (impedance matching).

The power dissipation $P_{ex}$ in the external load is shown in Figure 6(a), which becomes maximum when the external impedance is matched with the internal one by $R_{ex}=R_{in}$ at point A. When $R_{ex}\gg R_{in}$ at point B, most power is consumed by the internal small resistance. When $R_{ex}\ll R_{in}$ at point C, on the other hand, the external resistance draws most current output i but the net Joule heat $i^{2}R_{ex}$ is small due to the small resistance value. Considering the fact that a large resistance becomes a low loss component for a current source, it is a natural consequence that this equivalent circuit model behaves in a similar manner as the dash pods in the VDRG, because expression in Equation (34) takes the identical form as Equation (31) for χ. Therefore, χ in the VDRG model is thought to be a factor that determines the deliverable power to outside. Note that χ does not show the absolute power but it tells the leverage factor with respect to the power obtained by a non-resonance case.

In contrast, the ratio of the power consumption in $R_{ex}$ with respect to the total power consumption (sum of internal loss $P_{in}$and external loss $P_{ex}$) is expressed as

(35) $$\frac{P_{ex}}{P_{in}+P_{ex}}=\frac{\frac{1}{R_{ex}}}{\frac{1}{R_{in}}+\frac{1}{R_{ex}}}=\frac{1}{1+\frac{R_{ex}}{R_{in}}},$$

which also takes the identical form as Equation (31) for η. As also shown in Figure 6(b), the external load consumes exactly 50% of the total power when $R_{ex}=R_{in}$, at which the load power is also maximized. When $R_{ex}\gg R_{in}$ at point B, the most power is consumed in the internal resistance, while the external resistance becomes dominant when $R_{ex}\ll R_{in}$ at point C. Due to the same reason that the expression in Equation (35) is in the identical format of Equation (32) for η, it is considered to determine the ratio of the power delivered to outside. In other words, η can be used as an impedance matching ratio in the VDRG model.

It should be noted that the effectiveness can be improved to 100% but it does not always guarantee a large output power, as suggested by point C in Figure 6(a,b). A similar behavior can be seen in the VDRG model, as also suggested by point C in Figures 4(b) and 5(b). At this point, the effectiveness is nearly 100% but the output is small due to the poor mechano-electric coupling.

### 2.6. Design strategy for impedance-mismatched condition

In the discussion for Figure 4, we presumed that the oscillation of the suspended mass is not limited by the physical dimension of the harvester device even when the oscillation is enhanced by the large quality factor. In such a situation, large output can be pursued by equalizing the external resistance with the internal one, as usually referred to as the impedance-matching condition in an analogy to the electrical model, which can be seen in the previous section.

**Figure 7.**
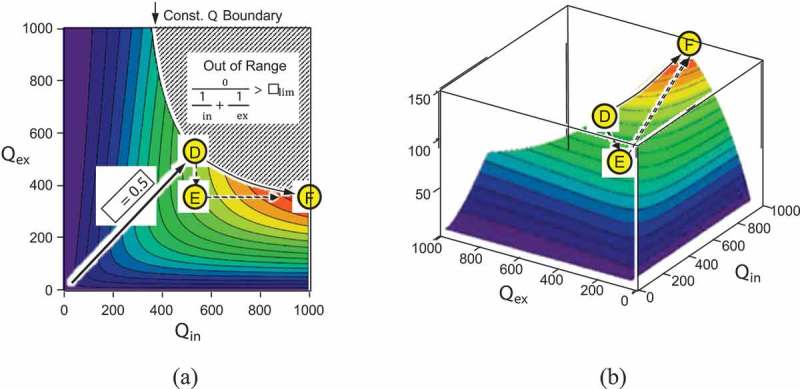
Power leverage factor χ calculated as a function of the internal and external quality factors when the oscillation amplitude is limited. (a) Contour plot and (b) 3D plot. Conditions for maximum deliverable power are found at point F, where the impedances are not matched.

The strategy to pursue a large output power may differ when the oscillation amplitude is limited due to the size of the harvester device. Figure 7(a) shows the contour plot of the power leverage factor χ, with a prohibited area that the amplitude exceeds the design limit as

(36) $$y_{0}\;Q=\frac{y_{0}}{\frac{1}{Q_{in}}+\frac{1}{Q_{ex}}}>X_{lim}.$$

In such a case, the impedance-matching point D does not deliver the maxim power but the device parameter should be tuned to seek for a high power at point F in Figure 7(b). One possible way to realize it is to shift the device performance along the equi-Q curve, which can also be processed by taking the detour route through point E to point F.

**Figure 8.**
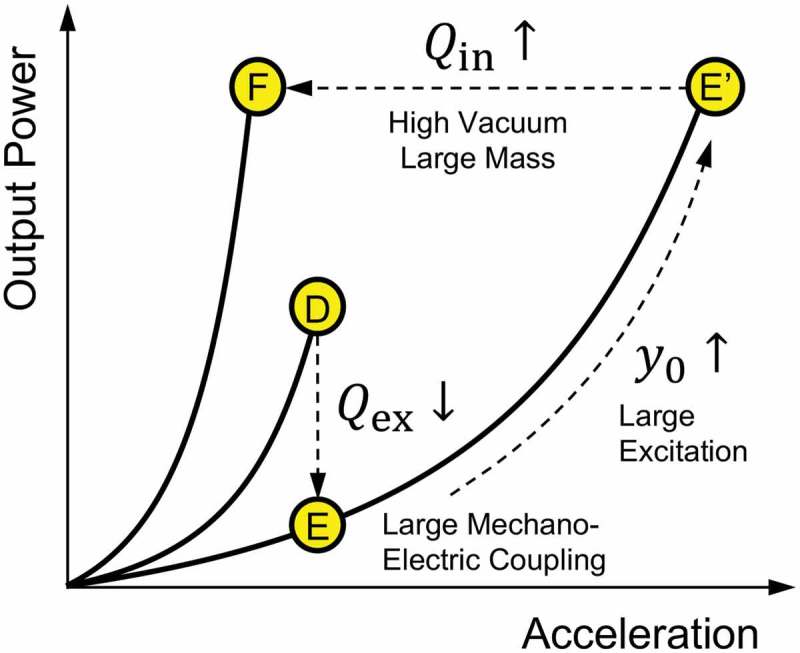
Schematic process to improve the output power when the oscillation amplitude is limited. Enhanced mechano-electric coupling (low $Q_{ex}$) will temporally lower the output (D to E) but it allows larger acceleration to be received without causing the amplitude clip (E to Eʹ). Equally large output is made possible at a lower acceleration when the internal quality factor is enhanced (Eʹ to F).

The state transition from point D to F is schematically illustrated in the power-acceleration curve shown in Figure 8. Point D shows a position that the output power remains small due to the oscillation amplitude clipped to the limit. In most cases, the amplitude clip occurs due to the poor mechano-electric coupling that fails to effectively damp the power into an electrical output. In such a case, the external loss is deliberately increased by enhancing the mechano-electric coupling, for instance, by which the mechanical stroke is efficiently converted into electrical power. By this modification, the output power at the same excitation acceleration becomes tentatively small to point E. However, one would use a larger acceleration to reach the oscillation limit marked by point Eʹ, where more power is delivered owing to the larger excitation power as well as the enhanced mechano-electric coupling. One would further enhance the output at point F, by increasing $Q_{in}$. From the analytical model $Q_{in}=\sqrt{m\;k}/c_{in}$, a large $Q_{in}$ is designed by adding a mass to m (while maintaining the resonant frequency) and by reducing the internal loss such as air viscosity or internal electrical resistance in the harvester device. In other words, the VDRG design at point F delivers the best effort output from a small excitation acceleration, given a device footprint and the source frequency.

### 2.7. Mechano-electric conversion mechanisms

**Figure 9.**
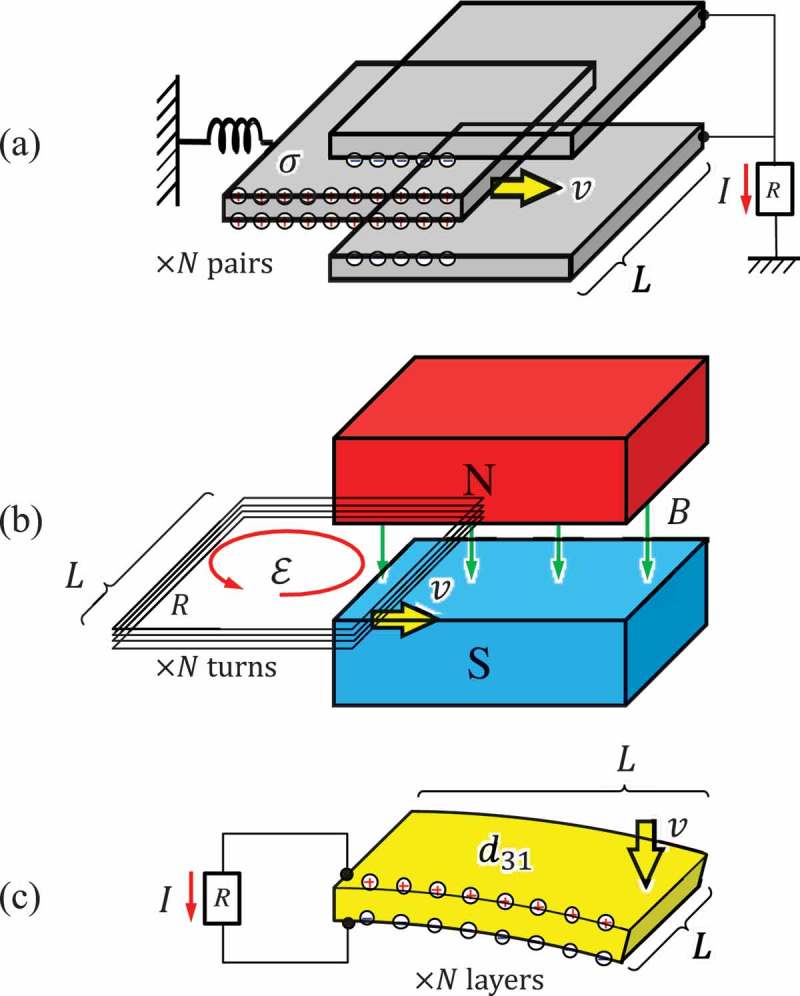
Schematic illustration of mechano-electric conversion principles. (a) Electrostatic induction, (b) electromagnetic induction, and (c) piezoelectric effect.

Having known that the quality factor $Q_{ex}$determines the output power of a VDRG, we then look into a way to design it in terms of the viscous damping coefficient $c_{ex}$. Figure 9 schematically shows three representative mechano-electric transduction mechanisms, namely, electrostatic induction, electromagnetic induction, and piezoelectric effect that are widely used in VDRGs. In this work, we use the dimension L shown in the figure to discuss the scaling effect of each mechanism. For simplicity, we assume that the mechano-electrically coupled power is consumed solely in the resistance R attached to the transduction mechanism.

Electrostatic induction is the most classic principle to generate electrical charges on the surface of conductive materials. In Figure 9(a), we show a movable electrode that is suspended in between the fixed electrode pair on the right-hand side. The movable electrode surface is coated with a thin layer of electrical insulating material that keeps a sheet of permanent electrical charge called ‘electrets.’ When the electret charge is uniformly distributed with an areal density of σ, the counter electrodes will build up a sheet of charges of the opposite polarity also at a rate determined by the insertion speed of the movable electrode v, and thus the electrostatic induction current is written as

(37) $$i=C_{1}\;\sigma \;L\;v,$$

where $C_{1}$ is a coefficient that is determined mainly by the ratio between the electrostatic capacitances formed in the electret film and in the air gap. The power dissipated in the resistance R is

(38) $$P_{ex}=i^{2}R={\left(C_{1}\;\sigma \;L\;v\right)}^{2}R.$$

Power consumption is also expressed in terms of the viscous damping coefficient $c_{ex}$ as $P_{ex}=c_{ex}\cdot v^{2}$, and hence, $c_{ex}$ for the electrostatic induction is written as

(39) $$c_{ex}={\left(C_{1}\;\sigma \;L\right)}^{2}R.$$

This result implies that the quality factor $Q_{ex}$ can be designed by engineering the value of $c_{ex}$. The electrode length, width, and gap length would be the primary design parameters, and the number of electrodes would be determined by considering the device footprint. There is no general expression for $C_{1}$ because it depends on the detail electrode geometry and the location of the electrets; nonetheless, $C_{1}$ can be made large for effective mechano-electric coupling by using a thin air gap between the electrodes.

Using a high-density electret is also an important task in the recent development of energy harvesters. The sheet density of electrets can be as high as 1 mC/m^2^ in silicon oxide film [19]. Due to the scaling ($L^{2}$) of $c_{ex}$, the output power quickly decreases by reducing the device dimensions, particularly when the capacitance plane is set in parallel with the device footprint. To overcome this constraint, the electrode gap of fine pitch is usually formed in the vertical direction with respect to the chip surface, by which the electrode pairs are increased and thereby $c_{ex}.$

Electromagnetic induction is the most popular principle of power generation in macroscopic scale power stations. When a coil traveling at a speed v is inserted in the gap of magnet as shown in Figure 9(b), the electromotive force caused by the electromagnetic induction is

(40) $$\varepsilon =-\frac{d\Phi }{dt}=-B\;L\;v.$$

The power consumed in the coil of resistance R is written as

(41) $$P_{ex}=\frac{\varepsilon^{2}}{R}=\frac{{\left(B\;L\;v\right)}^{2}}{R}.$$

Therefore $c_{ex}$ is extracted as

(42) $$c_{ex}=\frac{{\left(B\;L\right)}^{2}}{R}.$$

Compared with the electrostatic induction type, the design strategy for the electromagnetic VDRG is more straightforward, as one would use a strong permanent magnet for a large magnetic field B. The mechano-electric coupling is effectively enhanced by using a coil of many turns without increasing the device footprint. However, one should pay attention to the internal resistance of the coil that also increases with the turn.

Figure 9(c) shows a simplified schematic view of a piezoelectric energy harvester. There are many variations of analytical models for the piezoelectric type depending upon the combination of the direction of the stress T and the orientation of the piezoelectric axis of material. In this study, we simply use a piezoelectric cantilever with its tip bent down to induce the uniform tensile and compressive stresses on the top and bottom surfaces, respectively. The electric flux density D caused through the piezoelectric constant $d_{31}$ is written as

(43) $$D=d_{31}\;T+\varepsilon^{T}\;E.$$

where $\varepsilon^{T}$ is the dielectric constant and E is the electric field in the film. Presuming that the stress T is in proportional to the velocity v and that the flux density is mostly governed by T, then we write the output current as

(44) $$i=\frac{dD}{dt}L^{2}=C_{2}\;d_{31}\;v\;L^{2},$$

where $C_{2}$ is the coefficient that is determined by the rigidity of the cantilever as well as the boundary condition of the applied force. The power dissipated in the resistance R is

(45) $$P_{ex}=i^{2}R={\left(C_{2}\;d_{31}\;L^{2}\;v\right)}^{2}R$$

and the viscous damping coefficient is written as

(46) $$c_{ex}={\left(C_{2}\;d_{31}\;L^{2}\right)}^{2}R.$$

Note that the mechano-electric coupling is sensitive to the scale $L^{4}$, and hence, the output may quickly decrease when the VDRG is made to be small. Using multilayered piezoelectric material would be effective to moderate the scaling effect to $L^{3}$, which may however lose compatibility to the environmental vibrations because the bending structure becomes rigid and hence the resonant frequency becomes high.

In either case of electrostatic, electromagnetic, or piezoelectric type, the power conversion performance is proportional to the square of the velocity (and hence the frequency). Considering that the environmental vibrations are distributed in a relatively low-frequency range (100 Hz and lower), the conversion efficiency would have been poor if the vibration is directly coupled to a mass. When a resonant mechanism is used, on the other hand, the velocity of the mass is amplified by the *Q* factor, as theoretically shown by Equation (29), and the output power is accordingly enhanced.

## 3. Harvester devices

### 3.1. Electrostatic device

Electrostatic induction current is a generation principle that is directly caused by the mechanical motion of a conductor body in presence of the electrical fields. Permanent electrical charges or so-called electrets are used to build the electrostatic fields by trapping electrons or ions in electrical insulator materials. Recent studies use electrets made of amorphous fluorinated polymer CYTOP [20,21], Parylene-C [22], polyethylene [23], and silicon-based materials [24]. Electret is also used as a part of interposer that induces nonlinearity in the oscillation systems such that it would extend the bandpass for the incoming vibrations [24].

Honma et al. developed an electrostatic induction type VDRG by using a high-density electret formed on the surface of silicon micromachined comb-electrode oscillators [25]. Silicon oxide has been known to bear negative electrets in the form of SiO^−^ when impurities such as potassium ions are displaced from lattice network by the external electrical field applied at a high temperature [19].

**Figure 10.**
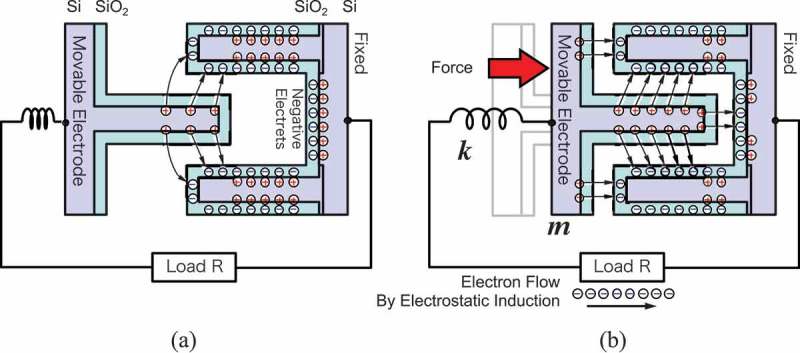
Detailed view of the electrostatic induction type VDRG. (a) The surface of the fixed electrode is coated with a negatively charged silicon oxide. The charges are bound with the positive charge at the silicon oxide/silicon interface at the rest position. (b) When the movable electrode is inserted into the fixed ones, the electrical flux between the negative and positive charges are rearranged in part, and the released electrons flow out, thereby converting the mechanical work into the electrical energy.

Figure 10 illustrates the electrostatic induction caused by the mechanical motion of the comb-shaped electrodes. The electrodes made of silicon are coated with a thin silicon oxide film (~1 µm), which has been negatively charged by the polarization process. The charges of opposite polarities are usually bound across the silicon/silicon oxide interface, and therefore the most electrical flux is confined within the fixed electrode. When the movable electrode is inserted into the gap, on the other hand, a part of such flux is redirected toward the counter electrode, thereby releasing the electrons that are forced to flow through the load resistance.

**Figure 11.**
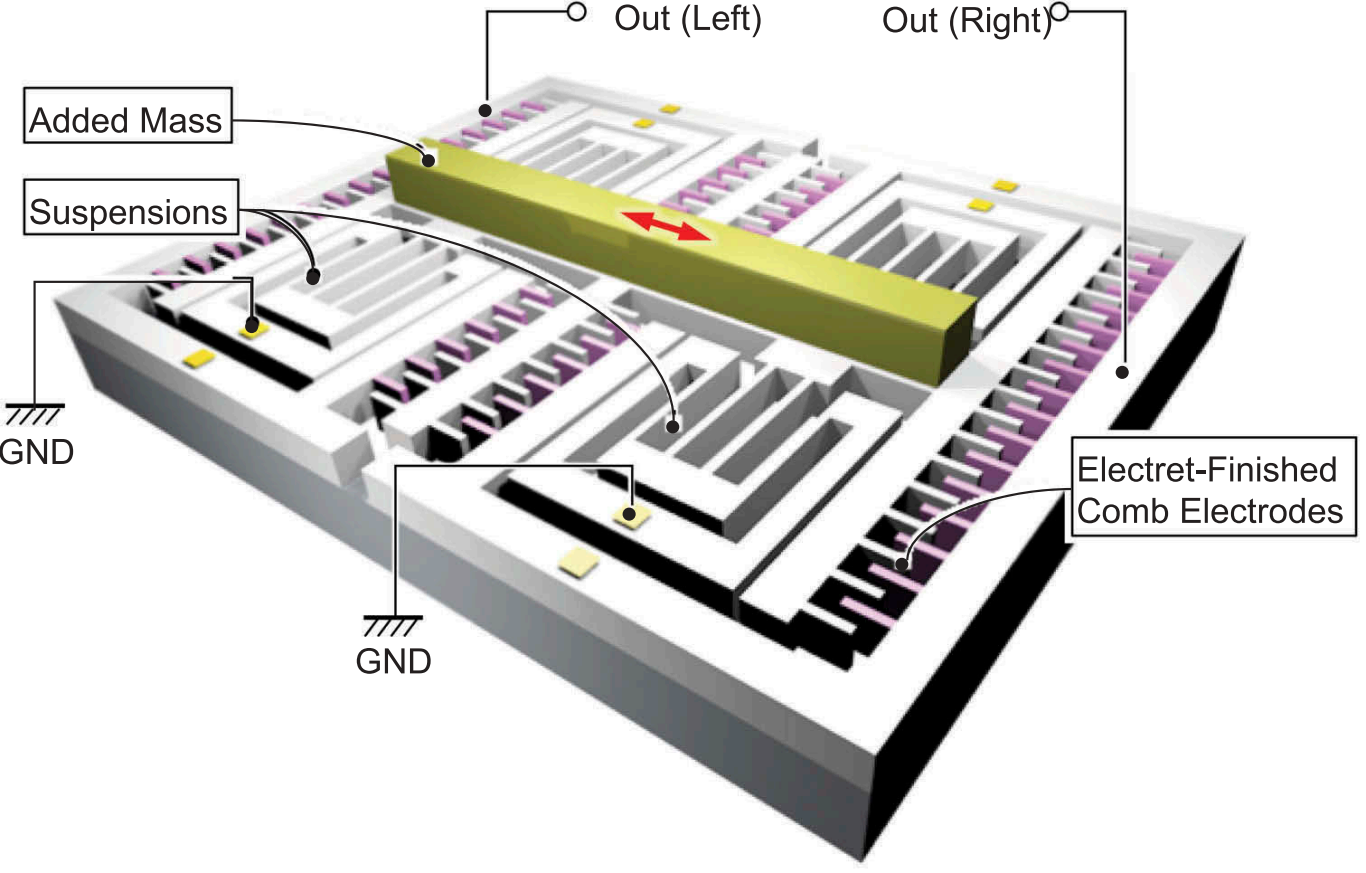
Schematic illustration of electrostatic induction type VDRG. High-density comb electrodes are used to increase the areal mechano-electric coupling. A mass is attached on the movable electrode to increase the mechanical quality factor.

The comb electrode mechanism in Figure 10 is usually implemented in the form of a vertical gap as schematically shown in Figure 11. The electrostatic capacitance is formed on the side walls of the microstructures, and hence, the surface-to-volume ratio is substantially increased rather than using the top surface of the chip as an electrode. In addition to this, the pitch and the gap of the comb electrodes are made to be as small as a few microns owing to the recent development of the semiconductor fabrication technologies such as high-aspect ratio reactive ion etching or deep-reactive ion etching (DRIE).

**Figure 12.**
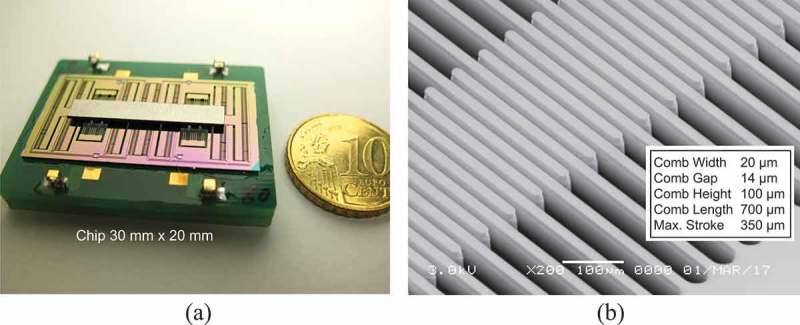
Example of electrostatic induction type VDRG made by the DRIE process on a silicon-on-insulator wafer. (a) Device photograph (30 mm × 20 mm) and (b) a close-up SEM view of the comb electrodes.

Figure 12 shows a photograph of the developed harvester and a close-up scanning electron microscopy (SEM) image of the comb electrodes. In this device, each comb is made to be 20 µm wide, 100 µm high, and 700 µm long to accommodate the maximum stroke of 350 µm. The narrowest gap between the opposing electrode is 13 µm. A total of 900 pairs of comb electrodes is integrated in a 30 mm × 20 mm chip.

**Figure 13.**
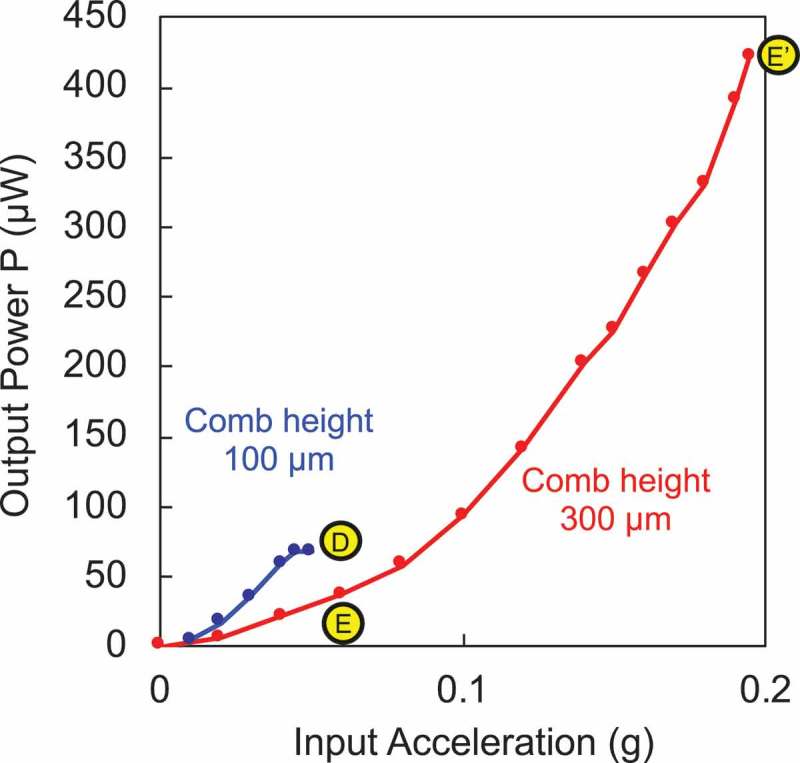
Experimentally measured output power from the electrostatic induction type VDRG (shown in Figure 12) as a function of the applied acceleration (1 g = 9.8 m/s^2^). Mechano-electric coupling is enhanced by using taller comb electrodes.

The resonant frequency of the device was designed at 125 Hz in order to couple the environmental vibrations that were experimentally characterized as a target energy source [25]. When the electret film was polarized to −200 V, the deliverable power was found to be only 70 µW at 0.05 g (1 g = 9.8 m/s^2^) as shown in Figure 13, and the power was clipped due to the limit of the mechanical stroke. When the comb height was changed to 300 µm, the output power at a given acceleration was found to decrease once but the deliverable maximum power was enhanced to almost 430 µW. The transition of the performance from point D through E to Eʹ took place as predicted by the curves shown in Figure 8. In other words, the mechano-electric coupling $c_{ex}$ was enhanced by increasing the electrode surface and hence the electret charge density per footprint.

### 3.2. Electromagnetic device

**Figure 14.**
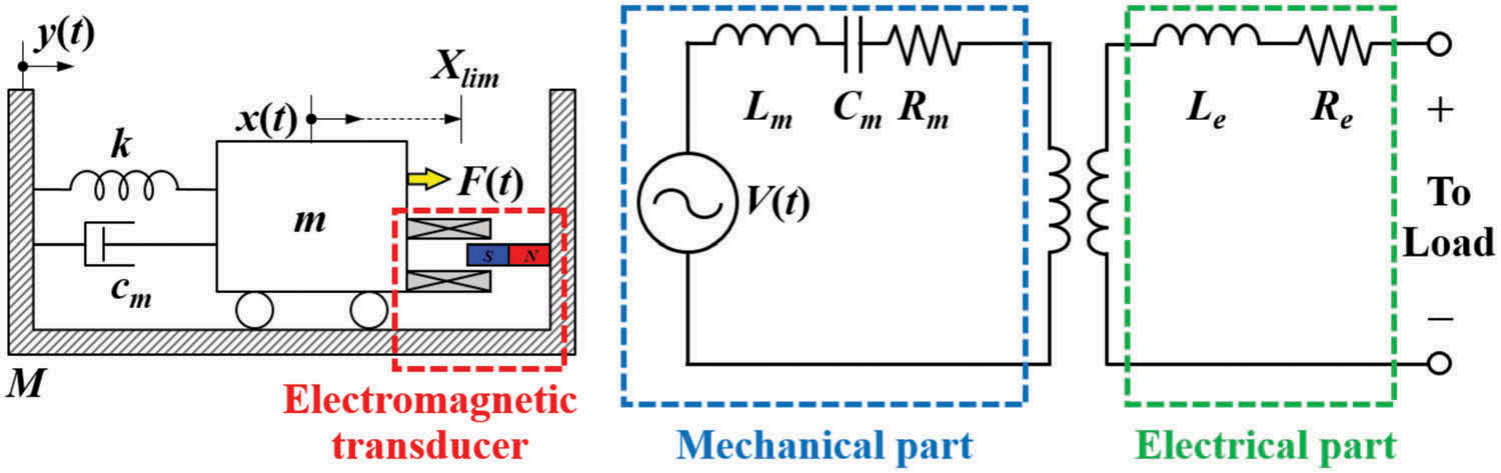
Typical lumped parameter representation of the spring-mass-damper-based electromagnetic vibration energy harvester (left) and equivalent circuit model (right).

The basic construction of the electromagnetic energy harvester is based on the relative motion of permanent magnets and coils, where the output voltage is proportional to the time variation of magnetic flux linking the coil according to the Faraday’s law. As shown in Figure 14, the most straightforward form of the electromagnetic vibration energy harvester utilizes spring-mass-damper system composed of the coil attached to spring members, such as cantilevers, located near the permanent magnet, and vice versa.

**Figure 15.**
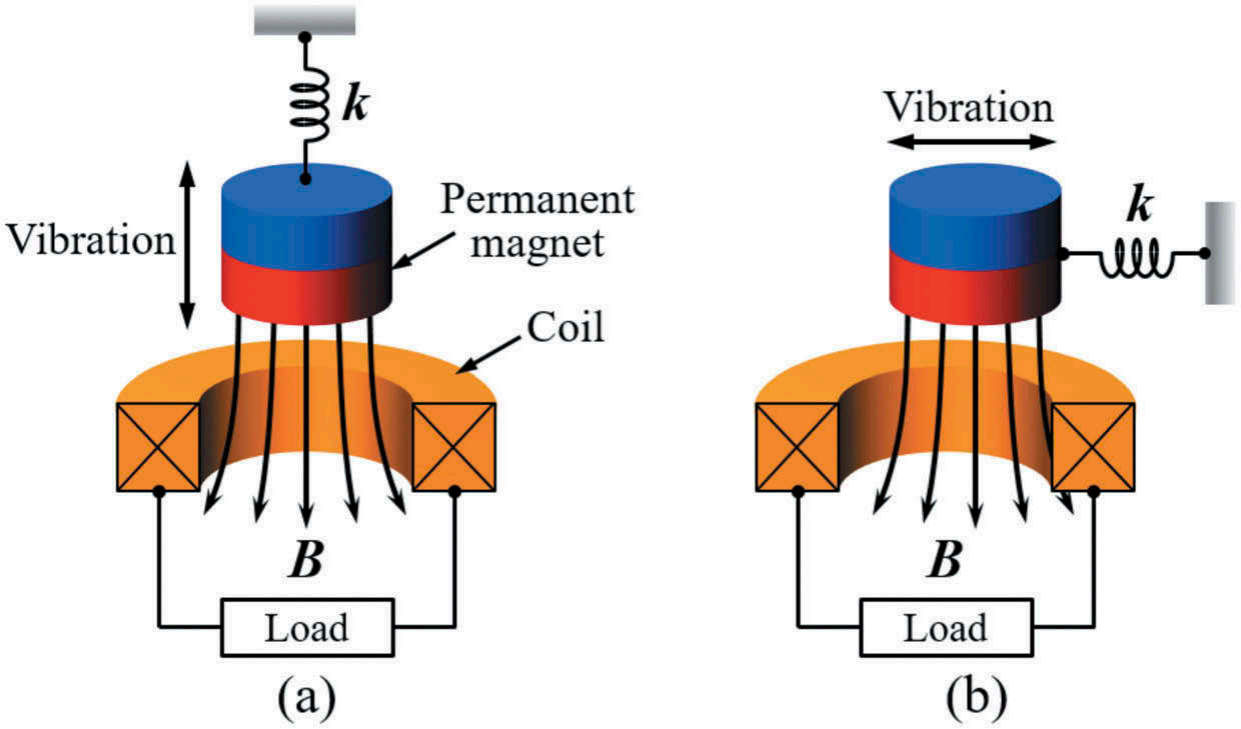
Two different arrangements for the relative displacement between the coil and magnet: (a) magnet moving parallel to the axis of the coil, (b) magnet moving perpendicular to the axis of the coil.

One of the unique features of the electromagnetic energy harvester is the flexibility in magnetic circuit design. Even the single coil and a magnet pair can be operated in two different configurations depending on the direction of the relative displacement of the two components as shown in Figure 15. Although the arrangement shown in Figure 15(a) is widely used in linear oscillatory generators, magnetic flux varies more sharply when the magnet moves perpendicular to the coil axis, which makes it a popular choice for most of the rotary generators as shown in Figure 15(b).

**Figure 16.**
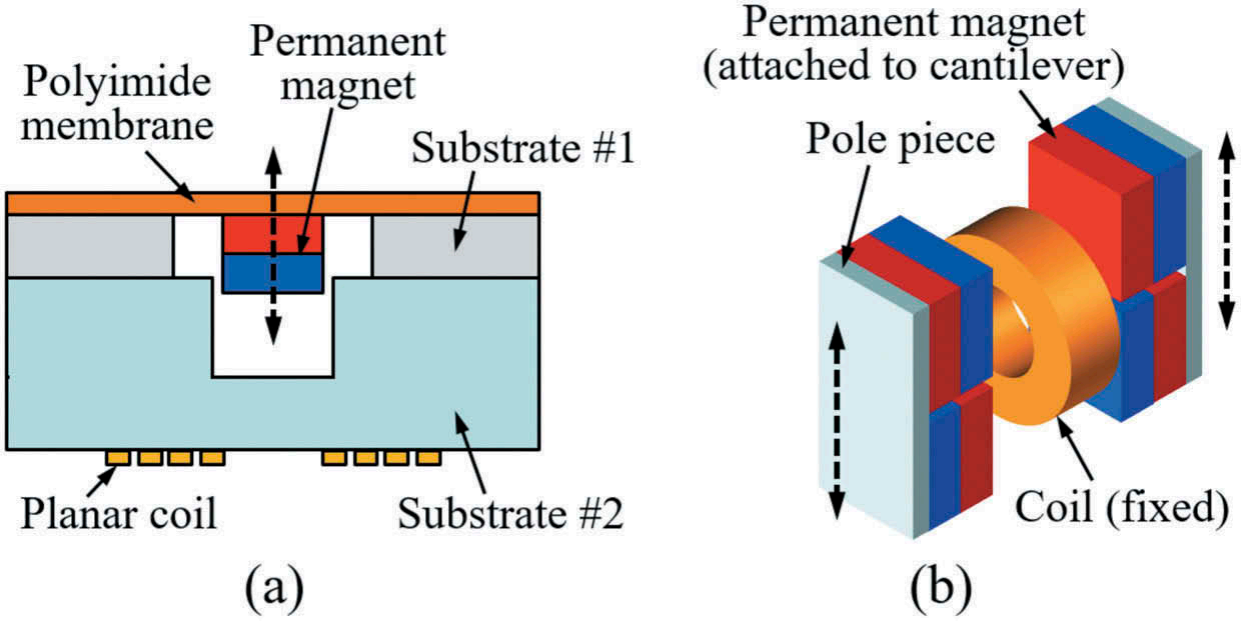
Electromagnetic energy harvesters with different magnet and coil arrangements: (a) magnet moving parallel to the coil axis [26], (b) magnet moving perpendicular to the coil axis [27].

As an example of the architecture shown in Figure 15(a), Williams et al. developed an electromagnetic generator by layering a substrate with a samarium–cobalt permanent magnet and a polyimide membrane to another substrate with a planar coil made of gold as schematically shown in Figure 16(a) [26]. An output power of 0.3 µW was generated at 4 kHz in a vacuum of 10^−5^ Torr. Beeby et al. developed a device with a permanent magnet assembly that moves in perpendicular to the axial direction of the coil as shown in Figure 16(b) [27]. Two pairs of magnets were attached to the cantilever, and the magnets were backed with pole pieces to generate a concentrated magnetic flux gradient. An output power of 46 µW was generated at 52 Hz. Examples in Figure 16 represent not only the two distinctive arrangements of magnet and coil but also demonstrate two different approaches for device fabrication. For macroscale and mesoscale devices fabricated with discrete components, high-performance rare earth magnets and high-density self-wound coils are widely used, which enables a wide variety of approaches to control the external quality factor $Q_{ex}$. In contrast, challenges have to be considered for successful miniaturization of the device, including the microfabrication of permanent magnet for high power electronics and increasing the number of coil turns. For microfabricated devices, increasing the internal quality factor $Q_{in}$ using vacuum packaging could be a useful option. To overcome the limitations in miniaturization and integration of the permanent magnet materials, Palmero et al. developed polymer/manganese–aluminum-based composite and filament, which can potentially be used in bonding and three-dimensional (3D) printing technologies [28], and Ewing et al. developed electrodeposition process to produce a 20-µm-thick cobalt–platinum layer on a silicon substrate [29].

**Figure 17.**
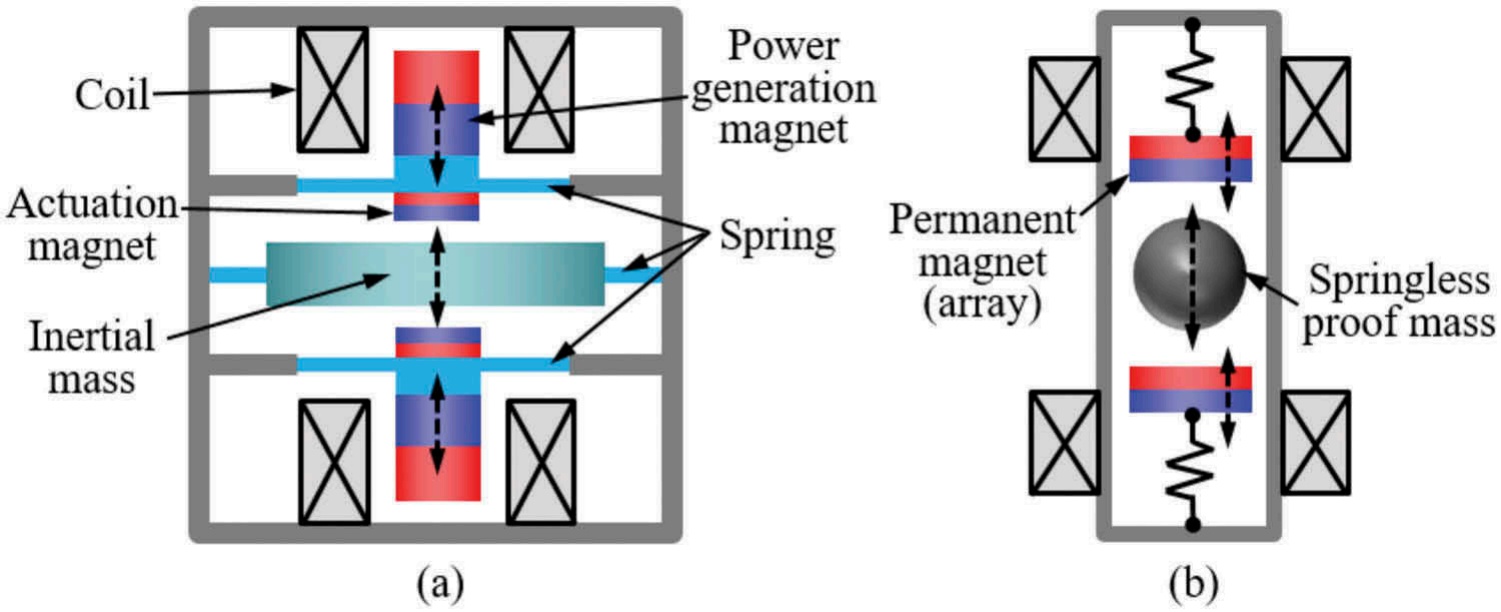
Frequency up-converted electromagnetic energy harvesters (a) using spring-supported inertial mass [30] and (b) using springless proof mass [31].

In addition to the efforts to optimize the related parameters of conventional spring-mass-damper system architecture, a new type of devices using nonconventional approaches has also been developed. Galchev et al. developed a frequency-up-converted power generator by using a magnetic plucking between a spring-suspended inertial mass and electromagnetic transducers which resonate at a higher frequency, as schematically shown in Figure 17(a) [30]. The device was designed to pursue low-frequency operation, broad bandwidth, and large output power at the same time, which is a challenging task for conventional resonant generators. At an input acceleration of 1 g at 10 Hz, 13.6 µW was generated, and a 3-dB bandwidth of 55 Hz was obtained. Hallim et al. used a springless mass inserted between the pair of magnets suspended to the both ends of a channel as shown in Figure 17(b) and achieved a frequency up-conversion [31]. An output power of 203 µW was generated in response to hand-shaken vibrations. *Q*-fold amplification of oscillation amplitude is not expected because the suspended inertial mass or springless poof mass in these approaches is not intended to resonate. However, relatively large masses can be used to lower the operating frequency range as well as increase the impact force to the electromagnetic transducers, which resonate at a frequency considerably higher than that of the input vibrations.

Various types of devices have been developed with a permanent magnet as a springless mass in a view to overcome the limitations of the conventional spring-mass-damper-based architecture and to utilize a low-frequency environmental vibration. In such cases, optimization of electromechanical coupling becomes more crucial as the resonant behavior of the proof mass cannot be utilized. Use of multidirectional coils and an array of magnets is a good example to pursue a higher changing rate of magnetic flux to multiply the output voltage. Bowers et al. [32] used a spherical magnet inside a cavity surrounded by a coil to harvest energy from human motion, which demonstrated time-averaged power densities of up to 0.5 mW/cm^3^. Chae et al. utilized an array of rectangular permanent magnets sliding in the lateral direction with ferrofluid lubricant, which generated 493 µW in 3 g vibrations at 13 Hz [33].

### 3.3. Piezoelectric device

**Figure 18.**
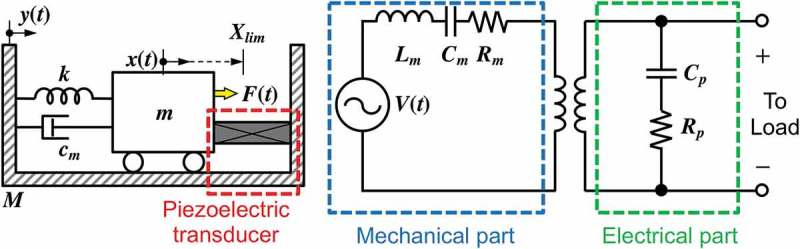
Typical lumped parameter representation of the spring-mass-damper-based piezoelectric vibration energy harvester (left) and equivalent circuit model (right).

Piezoelectric materials have been widely used in vibration energy harvesting due to relatively simple architecture and straightforward power generation mechanism. The piezoelectric effect takes place when piezoelectric materials are subjected to a mechanical strain, where the material is electrically polarized at a rate proportional to the applied strain. Conversely, these materials deform when exposed to an electric field by the inverse piezoelectric effect. Piezoelectric vibration energy harvesters are generally composed of a piezoelectric cantilever with a proof mass at the free end of the beam, as equivalently illustrated in Figure 18. Due to the inherent nature of the piezoelectric effect, surface area or footprint of the device determines the area available for piezoelectric material, and therefore the performance, unless the piezoelectric components are stacked.

**Figure 19.**
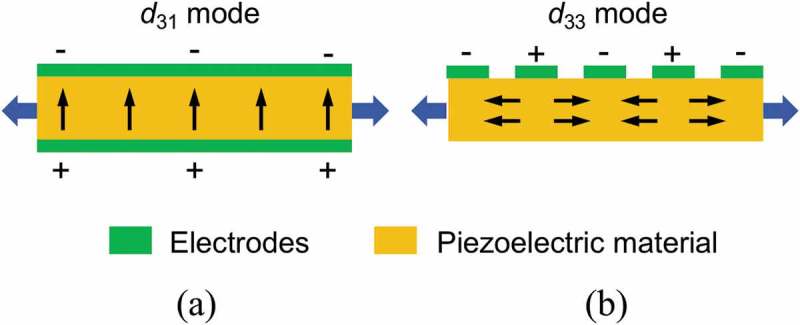
Poling direction of *d*_31_ mode (left) and *d*_33_ mode (right) piezoelectric materials [34].

Piezoelectric materials are widely available in various forms including single crystal (e.g. quartz), piezoceramic (e.g. lead zirconate titanate or PZT) [34,35], thin film (e.g. zinc oxide), polymeric material (e.g. polyvinylidene difluoride or PVDF) [36] and macro fiber composite [37–39]. Although thin film piezoelectric materials are available for microfabrication, piezoelectric constants of these materials are typically lower than those of the bulk PZT. Piezoelectric materials typically exhibit anisotropic characteristics. A *d*_31_ mode piezoelectric generator possesses top and bottom electrodes as shown in Figure 19(a), while *d*_33_ mode piezoelectric generator is fabricated with an interdigitated electrode (IDE), as schematically compared in Figure 19(b) [40–42]. Reports on the *d*_33_ mode piezoelectric energy harvesters are relatively rare compared to those using the *d*_31_ mode mainly because most piezoelectric films are c-axis oriented and partly because of the complexity in the IDE fabrication [40,43].

**Figure 20.**
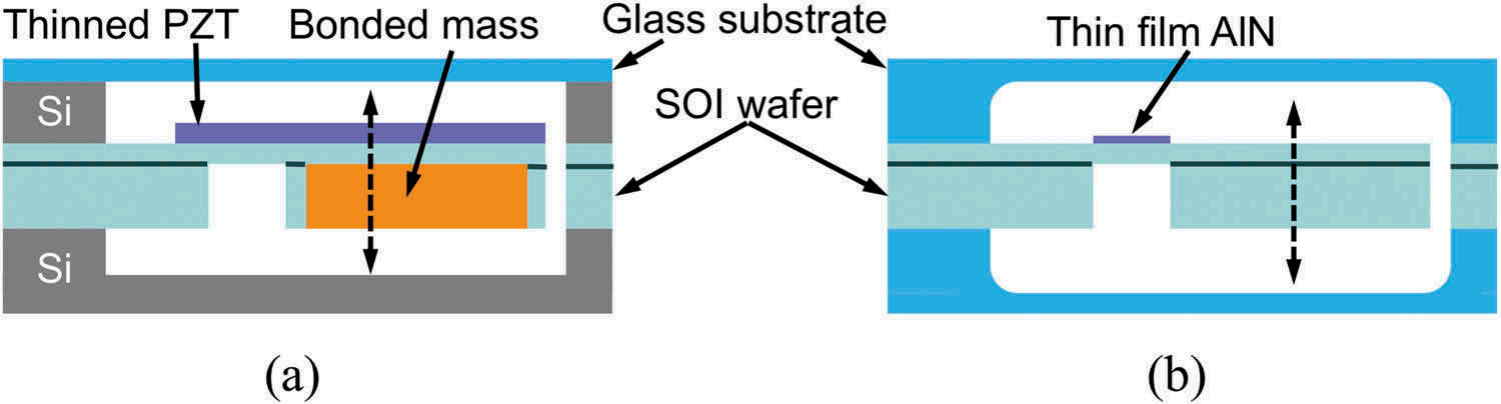
Schematics of the vacuum packaged piezoelectric energy harvester: (a) using thinned and bonded PZT [44], (b) using an AlN thin film [45].

Aktakka et al. developed a vacuum packaged piezoelectric MEMS energy harvester by bonding a thinned PZT and a tungsten proof mass to a silicon-on-insulator substrate as shown in Figure 20(a), which generated an output power of 205 μW at 1.5 g vibration at 154 Hz [44]. Kamel et al. developed a similar device as shown in Figure 20(b) by using an aluminum nitride (AlN) thin film as the piezoelectric material, which generated 24 μW at 0.8 g vibration [45]. Although PZT is the leading material in terms of the piezoelectric constant, AlN has also been used by considering the low dielectric constant and the ease of fabrication. Compared to the variation of electromagnetic devices, the device architecture for piezoelectric generators is limited to a few. Although the internal quality factor $Q_{in}$ can be improved by vacuum packaging, the overall performance of the device is still highly dependent on the property of the piezoelectric material used. Utilization of high-performance bulk PZT can be an option to increase the external quality factor $Q_{ex}$ at the cost of higher stiffness and increased fabrication complexity. Instead of utilizing bulk or thin film piezoelectric materials, Tsukamoto et al. developed bimorph piezoelectric vibration energy harvester with a flexible 3D meshed-core elastic layer sandwiched between two PVDF layers to improve the output power while lowering the resonance frequency. Output power of 24.6 μW has been obtained at 0.2 g vibration at 18.7 Hz [46].

**Figure 21.**
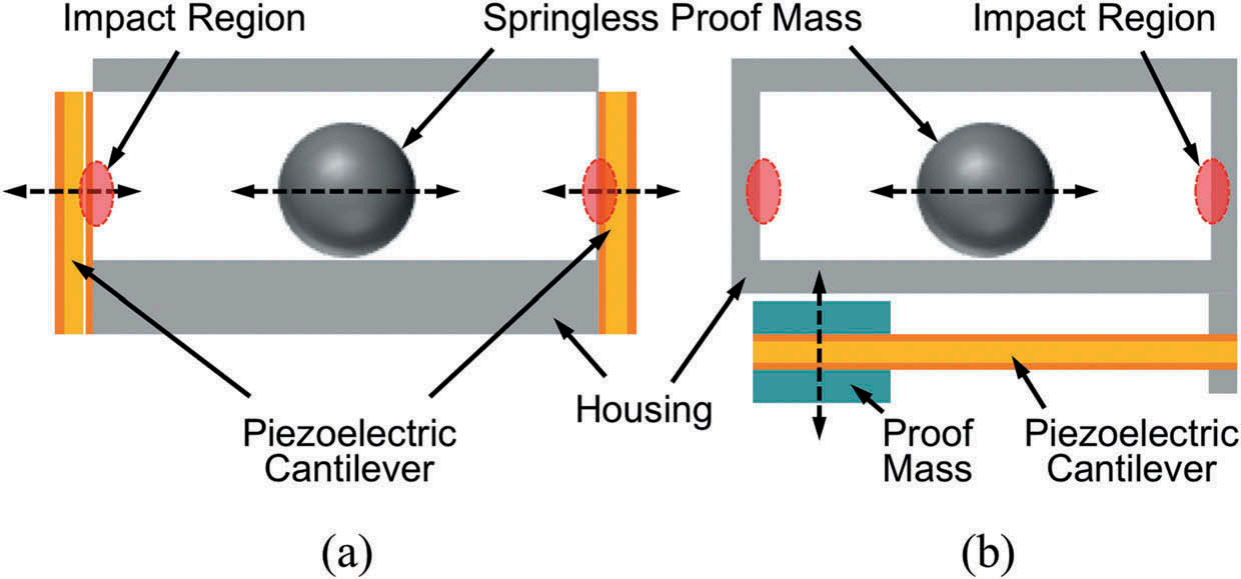
Schematics of the impact-based piezoelectric generator: (a) using the direct impact of a springless proof mass and piezoelectric cantilever [47,48], (b) using indirect impact [49].

One of the strategies to improve the performance of piezoelectric energy harvesters is to utilize an impact-induced vibration of piezoelectric materials. Renaud et al. have developed an impact-based piezoelectric energy harvester as shown in Figure 21(a) [47,48]. As the free-sliding metallic proof mass directly collides with the piezoelectric bimorph at the both ends of the channel, a high output power can be obtained at the cost of potential reliability risk due to catastrophic failure of the piezoelectric material. An output power of 600 µW was obtained from a linear motion of 10-cm amplitude at 10 Hz. As an alternative architecture, piezoelectric energy harvesting device with indirect impact mechanism, as schematically shown in Figure 21(b), has been proposed to avoid physical contact between the piezoelectric material and the springless proof mass, while maintaining the advantages of the impact [49]. In this architecture, the piezoelectric cantilever is forced to vibrate at the resonance frequency of the housing, which is considerably higher than that of the piezoelectric cantilever. Maximum peak-to-peak open circuit voltage of 42.2 V and average power of 633.7 μW were obtained for a 3-g acceleration at 17 Hz. With a device having parallel-connected piezoelectric materials, the maximum average power was increased to 963.9 μW for a 3-g acceleration at 18 Hz.

For the impact-based architectures shown in Figure 21, the frequency of the excitation accelerations is transformed into frequency twice as high due to the periodic impacts on both ends of the channel. In contrast to the direct impact-driven device, where the force of the proof mass is directly transformed into electrical energy, the vibration of the indirect piezoelectric cantilever is induced by the vibration of the housing. Therefore, the resonance frequency of the cantilever is determined by the geometry and material of the housing, where the design of the fixed end of the cantilever plays a critical role.

## 4. Benchmark test

**Figure 22.**
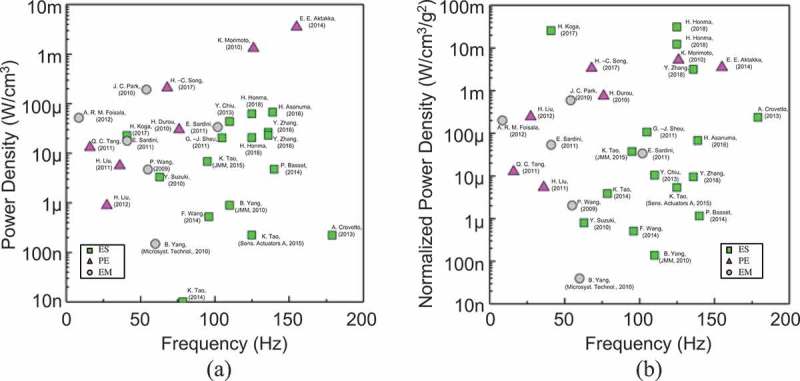
Benchmark comparison of VDRG performance in a low-frequency range (200 Hz or lower) for the environmental vibrations. (a) Power density comparison and (c) normalized power density comparison [13,50–72].

Figure 22(a) compares the VDRG performance in the frequency range for the environmental vibrations (200 Hz or lower). We use the volumetric power density (PD, µW/cm^3^), which is the output power divided by the device volume, as an index to compare various types of VDRG including electrostatic [20,50–61], electromagnetic [62–66], and piezoelectric [13,67–72] types reported in 2009 and later. Considering the application to the environmental vibrations, the performance becomes preferable when a benchmark dot is placed near the top-left corner. As the output power of VDRG is proportional to the square of excitation stroke as shown by Equation (28), we also use the normalized power density (NPD, µW/cm^3^/g^2^), which is the volumetric power density further normalized by the square of applied acceleration, as a function of the operation frequency as shown in Figure 22(b).

From Figure 22(a), one can tell that the piezoelectric type occupies an area of large PD. However, their NPD index in Figure 22(b) tends to shift downwards because of the relatively large rigidity of the mechanical structures that requires large excitation force (or acceleration) for operation. The electromagnetic and electrostatic types have almost the same distribution in the PD plot but the former has better position in the low-frequency range, plausibly because of the mass of the coil or magnet that lowers the resonant frequency; nonetheless, it also implies that an assembly process is required for a magnet and a coil for the electromagnetic type. Owing to the recent development of high-density electret materials, the best NPD performance is achieved by the electrostatic type. Note that Figure 22 mostly uses the operation resonant frequencies of the energy harvesters for the horizontal axis. Recent studies also use non-resonant type operation for extended bandwidth for incoming vibrations through the interposer mechanisms of nonlinear oscillation based on the mechanical bi-stability [24,73].

## 5. Conclusions

Reflecting the emerging business opportunities in the IoT market, energy harvester devices are increasing their importance as an autonomous power source. Amongst various environmental power sources, we picked up the vibrational energy harvester because vibrations are the most redundant power source that could be ubiquitously found around the clock. In this paper, we deduce the analytical model for the electrical output from a velocity-damped resonant type energy harvester and presented a methodology to tune the internal and external quality factors of resonance to maximize the output power given a source frequency, acceleration, and device footprint. Three major conversion mechanisms are studied to formulate the scaling effect of the mechano-electric transduction. Recent examples of these three types of energy harvesters are case-studied and compared in terms of the power density as well as the normalized power density. Considering the power demands of recent microelectronics, vibrational energy harvesters of 100 μW power level became feasible as commercial products. When targeting operation for higher power or at a lower acceleration, the analytical model presented in this paper would be useful for a reasoning of engineering parameters with the device performance.
